# Cognitive psychiatry in India

**DOI:** 10.4103/0019-5545.69224

**Published:** 2010-01

**Authors:** P. K. Dalal, T. Sivakumar

**Affiliations:** Department of Psychiatry, C.S.M. Medical University UP, (Erstwhile King George’s medical College), Lucknow - 226 003, India; 1 All India Institute of Medical Sciences, New Delhi, India

**Keywords:** Attention, bipolar disorder, brain injury, cognition, compulsive disorder, depression, executive function, memory, mini-mental status examination, obsessive compulsive disorder, Electroconvulsive therapy (ECT), psychotherapeutics, schizophrenia, substance abuse

## Abstract

Cognitive deficits have been shown to exist in various psychiatric disorders. Though most Indian studies pertaining to cognition have been replication studies, well designed original studies have also been conducted. This article traces the evolution of cognitive psychiatry in India. Cognitive research has huge potential in India and can help us unravel mysteries of the human mind, identify etiopathogenesis and facilitate treatment of psychiatric disorders.

## INTRODUCTION

### What is cognition?

Cognition is what enables humans to function in everyday life: Personal, social, and occupational. The ability to attend to things in a selective and focused way, to concentrate over a period of time, to learn new information and skills, to plan, determine strategies for actions and execute them, to comprehend language and use verbal skills for communication and self-expression, and to retain information and manipulate it to solve complex problems are examples of mental processes that are referred to as cognitive functions.

### Cognitive deficits

Cognitive deficits may result in the inability to:

1. Pay attention
2. Process information quickly
3. Remember and recall information
4. Respond to information quickly
5. Think critically, plan, organize and solve problems
6. Initiate speech

### Cognition and psychiatric disorders

Cognitive abilities have been shown to be impaired in patients with psychiatric disorders [Table 1].[1]

**Table 1:** Cognitive deficits seen in various psychiatric disorders

Psychiatric disorder	Areas in which cognitive deficits are seen
Schizophrenia	Working memory, verbal episodic memory, attention executive function, old learning, vocabulary, visual perceptual skills
Mood disorders	Attention, executive function, verbal memory
Obsessive compulsive disorder	visuospatial, visuoconstructional, non verbal (encoding and retrieval), executive function
Somatoform disorder	Semantic memory, verbal episodic memory and visuospatial tasks
Borderline personality disorder	Attention-vigilance, verbal learning, verbal memory, executive function
ADHD	Executive function, sustained attention, memory
Substance abuse	Attention, encoding new information, cognitive flexibility, problem solving

Cognitive deficits contribute to poor functional outcome and therefore have been suggested as treatment targets in schizophrenia and possibly in other psychiatric disorders as well. They have also been shown to be possible endophenotypes.

## METHODOLOGY

For this review, a web-based review of current and past issues of Indian journal of psychiatry was carried out with keywords cognition, cognitive, memory, attention, concentration, executive, neuropsychological, learning, amnesia etc. Publications in other journals were accessed through Google scholar and PUBMED search using the words India, cognition, cognitive, memory, attention, concentration, executive, neuropsychological, learning, amnesia etc in various combinations. Back references of articles were done and this yielded a few more articles. The topic of dementia has been left out as most of Indian studies have involved epidemiological aspects only. The studies have been presented in tabular form for the sake of convenience and ease in reading. “

### Evolution of cognitive psychiatry in India

The first study relating to cognition in Indian Journal of Psychiatry was published in the year 1966 by Murthy *et al*.[2] on the effect of ECT on intelligence and memory in schizophrenic patients. This was followed up by studies on cognition in cannabis users and other populations.

Indian studies have generally been carried out in small number of subjects and tried to replicate research findings from the West; they have studied cognitive deficits in patients with common psychiatric disorders without involving less common entities. Of late, studies have included parents, siblings[3] and offsprings[4] of patients and revealed that they too suffer from cognitive deficits, which is in line with western research. There is a paucity of studies involving the pediatric population.[5] Although most Indian studies have been cross sectional studies, long term follow-up[6] studies have also been done by researchers. Recent studies have shown correlation between cognitive deficits and work functioning, rehabilitation.[7,8]

The type of cognitive tests administered has evolved from simple tests like MMSE to more sophisticated computer based tests. However, more sophisticated tests are available only in select centers in the country. Earlier studies used western tests like Wechsler memory scale, Luria Nebraska neuropsychological battery etc. Over the past few decades, Indian tests like PGI battery of brain dysfunction[9] and NIMHANS neuropsychological battery[10] to assess cognition have been developed and have been widely used in later studies. Indian norms of western tests have also been derived recently.[11,12] Recently, Trivedi *et al*. (personal communication) translated MATRICS-CCB in Hindi. This is likely to provide a standardized way of assessing cognition in Indian patients and pave the way for clinical trials of molecules targeting cognition.

Early research work studied effect of ECT[2,13,14] and Lithium[15] on cognition. Recently, there have been human studies on the effect of herbal formulations[16,17] and piribedil[18] in patients of mild cognitive impairment. Notable work has been done on the effect of various drugs in ECS related amnestic deficits[19–23] in rats, which has helped to further our knowledge. However, there has been no significant work on the effect of commonly used psychotropic medications on cognition in psychiatric patients.

Research relating cognition to other biological research (like neuroimaging) to map neuroanatomical correlates has just begun.[14] There has been no significant work in areas of social cognition, which has received much attention in the international forum.

The results of most Indian studies have been in accordance with western work with a few notable exceptions like negative correlation between cognition and work performance in Indian schizophrenic patients which was hypothesized to be due to social factors.[8]

There has been a steady increase in the number of articles pertaining to cognition in Indian Journal of Psychiatry over the past decade. It is encouraging to note that work by Indian researchers has also been published in reputed international journals.[6,19,24–26] There have been editorials on the subject in Indian Journal of Psychiatry.[27] Marfatia award[4] and DLN Murthy Rao orations[1,20] have been delivered in ANCIPS for Indian research pertaining to cognition.

### Indian tests to assess cognition

The validated Indian tests available in the field are

1. NIMHANS neuropsychological battery:[10] Composed of tests taken from other standardized battery of tests, such as the Luria-Nebraska Neuropsychological battery.
2. PGI battery of memory dysfunction:[9] Includes 10 subtests including forward and backward digit spans, one minute delayed recall of a word list, immediate recall of sentences, retention of similar word pairs, retention of dissimilar pairs, visual retention, visual recognition, recent memory, remote memory and mental balance test.
3. Hindi cognitive test battery of “The Indo-US cross national dementia epidemiology study”:[28,29] Is based on the English language cognitive screening panel used by the MOVIES project. The MOVIES battery includes a brief global cognitive scale (general mental status), the mini-mental status examination (MMSE) and a set of other tests tapping several other cognitive domains.

NIMHANS neuropsychological battery has recently been validated for children.[30]

Hindi MMSE[28] was developed as part of the Indo-US cross-national dementia epidemiology study but has been shown to be different from MMSE.[31]

Normative data for Wisconsin card sorting test (WCST) for the Indian population was reported recently.[11] The study found highly significant differences between the means on almost all WCST scores among the western and Indian sample, except for the number of correct responses.

The scores of Trail making tests in Indian population have been shown to vary from published results from other cultural groups.[12]

Indigenous methods[32–34] have been developed by authors to assess cognition.

### Cognition and substance abuse

The studies on cognition and substance abuse are reviewed in table 2.

**Table 2:** Studies on cognition in substance abuse

Study	Sample	Tests for cognition	Result
Agarwal *et al*. 1975[35]	40 chronic Bhang users	Wechsler memory scale, Bhatia’s battery of intelligence test, Bender visuo-motor gestalt test	No memory disturbance in majority of cases IQ <90 in 25% of patients Cognitive disturbance in 20% patients on Bender-Gestalt test
VenkobaRao *et al*. 1975[36]	34 psychiatric patients with H/O ganja smoking >1 year	Attention span, The spiral after effect Digit forward and digit backward, logical memory, Paired associate learning, visual reproduction, Recognition-photographs and weight discrimination	Improvement in paired associate learning and photograph recognition. The only deleterious effect observed was on the judgment of weights under the influence of ganja.
Mendhiratta *et al*. 1978[24]	25 Charas smokers	Bender Visual-Motor Gestalt Test, Maudsley Personality Inventory short form, and digit span tests from the WAIS	Compared with controls, cannabis users were found to react more slowly, to be poorer in concentration and time estimation, and greater perceptuo-motor disturbance. The *charas* smokers were the poorest performers and also showed poor memory, lowered psychomotor activity, and poor size estimation.
	25 Bhang drinkers		
	25 controls		
Sabhesan *et al*. 1990[37]	11 alcohol dependent (DSM-III) head injured patients continuing to consume alcohol 11 alcohol dependent head injured patients abstaining from alcohol 11 non-alcoholic head injured patients	PGI memory scale	Head injured persistent alcohol abusers were the poorest in performance and abstinence was followed by a welcome change
Narang *et al*. 1991[38]	30 DSM III alcoholic patients	P.G.I. battery of brain dysfunction	Significant relationship between cognitive impairment and duration of alcohol use
Mendhiratta *et al*., 2006[6]	Follow up study of Mendhiratta *et al*., 1978[24] after 9-10 years;	Bender Visual-Motor Gestalt Test, Maudsley Personality Inventory short form, and digit span tests from the WAIS	Significant additional deterioration in case of the users on digit span, speed and accuracy tests, reaction time and Bender visuo-motor gestalt test as compared to controls
	11 bhang users		
	19 charas smokers		
	15 controls could be followed up		
Saraswat *et al*. 2006[39]	30 alcohol dependent patents 15 controls	Trail making test Stroop test	Patient group performed poorly in all the tests
			Duration of abstinence over past one year correlated with performance on Stroop test
Silva *et al*. 2007[4]	25 high risk offspring of treatment seeking patients with alcohol dependence; 25 low risk offsprings of volunteers	Visual evoked response potential	Strong association between lower P300 amplitudes over frontal brain areas and an excess of externalizing behavior in high risk offsprings

## COGNITION AND SCHIZOPHRENIA

The studies on cognition and schizophrenia are reviewed in table 3.

**Table 3:** Studies on cognition in schizophrenia

Study	Sample	Tests for cognition	Result
Nizamie *et al*. 1992[40]	40 schizophrenic (DSM III) patients, 30 brain damaged patients and 30 normal controls	Luria Nebraska neuropsychological battery	Schizophrenic patients perform better than brain-damaged but had poor performance than in comparison to normal controls
Ananthanarayanan *et al*. 1993[33]	24 remitted schizophrenics, 25 currently ill neurotic depressives (ICD-9)	Computer based tests for visual information processing: Simple reaction time, choice reaction time, forced choice span of apprehension test	Remitted schizophrenics performed poorly on all these measures as compared to neurotic depressives
Mandal *et al*. 1999[41]	12 schizophrenics (DSM-III R) each with predominantly positive and negative phenomenology; 12 healthy controls	‘Recognition of Emotion’ sub-test of the Penn Facial Discrimination Task	Schizophrenic patients with negative symptoms exhibited a generalized emotion- recognition deficit. Schizophrenic patients with positive symptoms showed a deficit in recognition of ‘sad’ emotion
Mishra *et al*. 2002[42]	60 schizophrenic patients (ICD-9)	Luria Nebraska neuropsychological battery	Pattern of performance in tests indicated possibility of combined cerebral dysfunction, more towards left hemisphere functions
Sabhesan *et al*. 2005[43]	31 schizophrenic patients (ICD-10)	Executive functions assessment schedule, trail making test, Raven’s matrices, fluency tests	Patients had varying degrees of involvement of different dimensions of executive function tests. There was inverse relationship to TMT and a positive correlation with Raven matrices and fluency tests.
Das *et al*. 2005[44]	15 chronic schizophrenic patients (DSM-IIIR) 15 controls	Card Sort, Visual continuous performance task, Stroop test, Spatial task	Positive correlation between negative symptoms and neurocognitive functions especially card sort test Negative correlation between these two parameters and REM latency
Srinivasan and Thara *et al*. 2005[45]	100 chronic schizophrenic (DSM-IV) patients and 100 normal controls	Tests from Wechsler memory scale, Wechsler adult intelligence scale, San Diego neuropsychological test battery, NIMHANS	Schizophrenic patients performed poorly on all cognitive tests in comparison to normal controls. Cognitive deficits were related to gender, education, age, duration of illness, and presence of positive and negative symptoms.
Srinivasan *et al*. 2005[8]	88 chronic schizophrenic (DSM-IV) patients	Tests from Wechsler memory scale, Wechsler adult intelligence scale, San Diego neuropsychological test battery, NIMHANS neuropsychological battery	Cognitive deficits did not relate significantly to current employment status or to level of performance at work. Negative symptoms predicted employment status, and poor social functioning predicted poor work performance. It was speculated that social factors were underlying the high level of work functioning in this group despite cognitive deficits.
Malhotra *et al*. 2006[5]	14 childhood onset schizophrenia (COS) patients (ICD-10 DCR)	Wisconsin card sorting test	COS patients have difficulty in executive functioning Deficits similar to those of adult schizophrenia
Krishnadas *et al*. 2007[46]	25 schizophrenic (DSM-IV) patients in remission and 25 normal controls	Tests from PGI battery of memory dysfunction, NIMHANS neuropsychological battery, Rey-Osterrieth complex figure test, Frontal Assessment Battery.	Patients with schizophrenia showed significant deficits on tests of attention, concentration, verbal and visual memory and tests of frontal lobe/executive function as compared to normal controls. No relationship was found between age, duration of illness, number of years of education and cognitive function. No statistically significant relationship between cognitive function and scores on the disability scale.
Suresh Kumar *et al*., 2008[7]	34 patients of DSM-IV chronic schizophrenia; 40 patients with same diagnosis not attending vocational rehabilitation	MMSE	Cognitive functioning had positive correlation with occupational role in the rehabilitated group and negative correlation in the rehabilitated group. Social functioning had negative correlation with cognitive symptoms in patients without rehabilitation.
Trivedi *et al*. 2008[47]	36 non-affected first degree full biological siblings of schizophrenic (DSM-IV) patients and 36 controls	Wisconsin’s Card Sorting Test, Spatial Working Memory Test, Continuous Performance Test	Sibling group had substantial cognitive deficits as compared to control group. Siblings from multiples families (>1 schizophrenic patient in a family) performed poorer as compared to simple families.
Bhatia *et al*. 2009[3]	172 schizophrenic and schizoaffective patients (DSM-IV) and their parents (n =196) ; 120 controls	Trail making test	Cases as well as their parents showed more cognitive impairment than controls on the TMT

## COGNITION AND BIPOLAR DISORDER

The studies on cognition and bipolar disorder are reviewed in tables 4 and 5. Khanna *et al*. (1991)[48] described findings on Luria Nebraska neuropsychological battery in a 12-year-old boy with mania following encephalitis. The patient showed perseveration on rapid sequential hand movement, graphaesthesia items, extemporaneous speech and spontaneous writing. Attention problems and a tendency to answer impulsively and randomly was seen on spatial orientation items (Clock reading and Raven’s items), in counting numbers or days of week backwards and in memorizing a series of seven words. Interference procedures diminished his ability to remember. He had gross problems in arithmetic. Abstract reasoning was poor. Sequential thinking and planning skills were poor. After three months treatment with haloperidol, the patient showed some impairment in memory and intelligence though none of the clinical and summary scales were above the critical level.

**Table 4:** Studies on cognition in bipolar disorder

Study	Sample	Tests for cognition	Result
Taj *et al*. 2005[49]	30 bipolar disorder patients in remission 30 normal subjects	Digit symbol test, Trail making test part A and B, Verbal fluency test, Digit span forward and backward test, Logical memory test, Paired association learning test, Visual design reproduction test	Patients with bipolar disorder, in remission, have neuropsychological impairment in attention, memory and executive functioning
Trivedi *et al*. 2008[50]	15 euthymic bipolar 1 patients 15 controls	Wisconsin’s Card Sorting Test,	Euthymic bipolar patients showed significant deficits in executive functions.
		Spatial Working Memory Test,	
		Continuous Performance Test	
Trivedi *et al*. 2008[51]	10 first-degree apparently healthy relatives of patients with bipolar disorder 10 controls	Wisconsin’s Card Sorting Test,	As compared to the control group, performance of the relatives on tests for executive functions and vigilance was significantly poorer.
		Spatial Working Memory Test,	
		Continuous Performance Test	
Sareen *et al*. 2009[52]	25 first degree non affected full biological siblings of bipolar affective disorder patients 25 controls	Wisconsin’s Card Sorting Test,	The sibling group performed poorly on cognitive domains studied as compared to controls. Siblings from multiplex families performed poorly in some domains of executive function.
		Spatial Working Memory Test,	
		Continuous Performance Test	

**Table 5:** Studies comparing cognition in schizophrenia and bipolar disorder

Study	Sample	Tests for cognition	Result
Trivedi *et al*. 2006[53]	15 stable maintained schizophrenia (DSM-IV) patients; 15 euthymic bipolar-1 (DSM-IV) patients; 15 controls	Wisconsin’s Card Sorting Test,	Stable schizophrenia patients performed poorly on all the neurocognitive parameters as compared to both control and bipolar euthymic patients.
		Spatial Working Memory Test,	
		Continuous Performance Test	
Pradhan *et al*. 2008[54]	48 euthymic bipolar (ICD-10) patients; 32 schizophrenia (ICD-10) patients in remission; 23 normal controls	Wisconsin’s Card Sorting Test (WCST), Trail making test-B, Controlled words association test, PGI memory scale, Bhatia battery of performance tests of intelligence-Short scale, Bender visual motor Gestalt test, Trail A test	When compared to controls, both bipolar disorder and schizophrenia patients were significantly impaired on different tests of executive function, memory, IQ and perceptuomotor functions. Schizophrenic patients consistently performed worse than bipolar disorder patients but the difference was not statistically significant.

## COGNITION AND DEPRESSION

The studies on cognition and depression are reviewed in table 6.

**Table 6:** Studies on cognition in depression

Study	Sample	Tests for cognition	Result
Tandon *et al*. 2002[55]	50 depressed (ICD-10) patients	Wisconsin’s Card Sorting Test (WCST)	Depressed patients demonstrated poor performance on WCST. More severe illness was associated with greater impairment in executive functioning
	30 controls		

## COGNITION AND OCD

The studies on cognition and OCD are reviewed in table 7.

**Table 7:** Studies on cognition in OCD

Study	Sample	Tests for cognition	Result
Tarafder *et al*. 2006[56]	20 OCD patients (DSM-IV-TR)	Wisconsin card sorting test	Impairment of executive functions in patients
	20 controls		
Trivedi *et al*. 2008[57]	30 OCD (DSM-IV) patients and 30 controls	Wisconsin’s Card Sorting Test,	OCD patients perform significantly worse on cognitive measures than controls
		Spatial Working Memory Test,	
		Continuous Performance Test	

### Cognition in brain injured patients

The studies on cognition in brain injured patients are reviewed in table 8. Sharma *et al*. (1981) first reported the clinical usefulness of Luria Neuropsychological Investigation (LNI) on a group of brain damaged patients. Nizamie (1983) used LNI on a group of brain damaged patients. A number of studies since have been carried out (Nizamie *et al*., 1988; Panda, 1988; Sasi, 1989; Srivastava, 1989; James, 1990; Khanna *et al*., 1991; James *et al*., 1991) using the original version of Luria Nebraska Neuropsychological Battery.[40] There has been case reports that cognitive rehabilation improves cognition and functional ability in patients with reversible and progressive brain injury.[60]

**Table 8:** Studies on cognition in bran-injured patients

Study	Sample	Tests for cognition	Result
Chatterjee *et al*. 1979[58]	37 moderately severe head injury patients	Bender gestalt test,	Statistically significant improvement in cognitive functions over time
	Followed up at 6 months and 1.5 years	Wechsler memory scale,	
		Progressive matrices	
Sabhesan *et al*. 1990[59]	18 month follow up of 61 head injury patients	PGI memory scale	Patients with acceleration injuries showed a poor performance in comparison to those with contact injuries. Memory was found to be related to indices of severity of injury, particularly post traumatic amnesia (PTA).
Sabhesan *et al*. 1990[37]	11 alcohol dependent (DSM-III) head injured patients continuing to consume alcohol, 11 alcohol dependent head injured patients abstaining from alcohol and 11 non-alcoholic head injured patients	PGI memory scale	Head injured persistent alcohol abusers were the poorest in performance and abstinence was followed by a welcome change

### Cognition in other patients

The studies on cognition in other patients, cognition and ECT, psychotherapeutic studies on cognition and cognitive experiments on animals are reviewed in tables 9, 10, 11 and 12 respectively.

**Table 9:** Studies on cognition in other patients

Study	Sample	Tests for cognition	Result
Hackett *et al*. 1998[61]	26 children with epilepsy	Modified Oseretsky Test of motor incoordination	Patients performed as well as controls on the non-verbal test, but performed worse on tests of vocabulary and reading, suggesting a specific disadvantage in the area of education
	4 controls	Raven’s Colored Progressive Matrices Malayalam Vocabulary Test Malayalam Graded Reading Test	
Edwin *et al*. 1999[62]	20 HIV seropositive symptomatic patients	Delayed response ability, differences and similarities test, ideational fluency, paired associate word learning test, logical memory, digit forward, digit backward, digit symbol substitution test, bender gestalt test, complex figure learning test, color cancellation test, paced auditory serial addition test, Koh’s block design test, trail making test-part A, Benton visual retention test	Broad spectrum of impairments occur n HIV seropositive patients; impairments of various functions occur at different phases of the illness
	20 HIV seropositive asymptomatic patients		
	20 HIV seronegative controls		
Mishra *et al*. 2002[63]	30 epileptic patients	Luria Nebraska neuropsychological battery	Performance level and pattern of epileptics were significantly different from normal controls on all the parameters.
	30 controls		
Sahu *et al*. 2005[64]	30 women exposed to methylisocynate (MIC) in Bhopal gas disaster 30 control women	PGI battery of brain dysfunction	MIC exposed women had significant neurocognitive dysfunction in some specific areas as compared to control
	30 control women		
Srinivasan *et al*. 2005[65]	130 patients aged ≥60 years of age admitted for medical or surgical treatment	MMSE, Global rating of memory decline, Global rating of Intellectual decline	Patients ≥70 years of age with acute medical problem are most likely to have cognitive problems
Pawar *et al*. 2006[66]	30 patients undergoing renal transplant	Wechsler adult performance intelligence scale, Luria Nebraska neuropsychological battery	Successful renal transplant is associated with improvement in depression, IQ and life satisfaction

**Table 10:** Studies on cognition and ECT

Study	Sample	Tests for cognition	Result
Murthy *et al*. 1966[2]	15 Schizophrenic patients on ECT	Alexander scale of intelligence, Wechsler memory scale	No significant impairment of Intellectual efficacy and memory function due to ECT Improvement in the intelligence scores and a quick recovery after ECT course, of memory functions to their pre-ECT level, appear to be pre-conditions for a good prognosis on ECT in schizophrenics
Shah *et al*. 1974[13]	10 depressed patients on unilateral dominant and unilateral non-dominant ECT each	Wechsler memory scale	No significant difference in mean scores of memory scales in both unilateral dominant and non-dominant ECT
Kunigiri *et al*. 2007[14]	15 right handed patients with major depressive patient with melancholic features	Orientation battery test, Trail making test-form A, Wechsler memory scale, Benton visual retention test	Disorientation more pronounced after 5^th^ ECT Significant memory impairment following ECT

**Table 11:** Psychotherapeutic studies on cognition

Study	Sample	Tests for cognition	Result
Singh *et al*. 1982[15]	30 manic depressive illness patients (Feighner criteria for primary affective disorder) on Lithium and 30 healthy controls	Bhatia’s battery of intelligence tests, Wechsler memory scale	No impairment in memory functioning in patients on Lithium
Andrade *et al*. 1998[16]	22 patients of age related cognitive decline on *memorin* and 23 patients on placebo.	Complex passage test, complex figure, block design test, Digit symbol substitution test, Selective reminding test	In the *Memorin* group, on most tests significant improvement in performances were observed after treatment; improvement in many of the memory tasks was, however, confined to males
	*Memorin* is a herbal formulation derived from Mandookaparni, Jatamansi, Yashtimadhu, Shankapushpi and Smruti Sagar; 3 months duration study		
Nagaraja *et al*. 2001[18]	19 patients of mild cognitive impairment on piribedil and 8 patients of mild cognitive impairment on placebo; 90 days study	MMSE	Patients with mild cognitive impairment had improvement in global cognitive function when treated with the dopamine receptor agonist piribedil
Raghav *et al*. 2006[17]	18 patients of age associated memory impairment on standardized *Bacopa monniera* extract (SBME) and 17 patients on placebo; 12 weeks study	Tests of Wechsler memory scale	SBME produced significant improvement on mental control, logical memory and paired associate learning
Jaykaran *et al*. 2009[67]	31 patients with depression on fluoxetine; 1 month study	PGI memory scale, six letter cancellation test	Patients show significant improvement on cognitive functions

**Table 12:** Cognitive experiments on animals

Study	Sample	Tests for cognition	Result
Sethi *et al*. 1983[68]	15 male Albino rats on Lithium carbonate and 15 male albino rats as control	Brightness discrimination reaction	Lithium carbonate influences long term memory in animals

In separate studies, Andrade *et al*. found that the herbal formulations, Mentat and Memorin effectively attenuated anterograde and retrograde amnestic effects of electroconvulsive shocks (ECS). An aqueous extract of Shankpushpi (*Evolvulus alsinoides*) conveyed no cognitive benefits to rats which received ECS. In similar experiments, Brahmi (*Bacopa monniera*) and mandookaparni (*Centella asiatica*) alone or in combination did not facilitate pe-ECS learning but attenuated ECS-induced anterograde and retrograde amnesia.[21]

In separate experiments, Andrade showed that Verapamil, Felodipine, sodium nitroprusside[22], phenylephrine,[19] mifepristone, indomethacin[69] and celecoxib attenuated ECT induced retrograde amnesia.[20] The theoretical explanations and possible implications have been discussed by Andrade in his DLN Murthy Rao oration.[20]

## CONCLUSION

A commendable effort has been put in by Indian workers in the field of cognitive sciences despite dearth of psychiatric centers, research facilities and resources in India. Andrade cited lack of funds as the reason due to which translational research to examine safety and efficacy of herbal formulations in humans who are treated with ECT for psychiatric disorders could not be done.[20]

Given the many constraints that exist in our setup, sheer diligence and innovativeness at times has led to publication of studies with very sound methodology.

Cognitive research has huge potential in India and can help us unravel mysteries of the human mind, identify etiopathogenesis and facilitate treatment of psychiatric disorders. We require an earnest endeavor on the part of mental health professionals and greater support from Government agencies, International organizations, pharmaceutical companies and private foundations for further progress in this exciting field.
